# Well Done, Nursing Staff! Next Please

**Published:** 1916-08-05

**Authors:** 


### Well Done, Nursing Staff! Next Please.

*To the Editor of* The Hospital.

Sir,—It may interest your readers to learn that the nursing staff of the 1/5th Northern General Hospital, R.A.M.C. (T.), Leicester, have subscribed amongst them £50 14s. to endow a bed for one year at Roehampton.

The various services comprising the unit are as follows: T.F.N.S., A.P.M.C., St. J.A.B., B.R.C., and all of them are represented. We venture to hope that other units may help in the support of this splendid place, and to suggest that a visit to it cannot fail to rouse great interest.—Believe me, yours faithfully,

A Territorial Sister.

